# Theoretical Boundary Conditions of Partner Buffering in Romantic Relationships

**DOI:** 10.3390/ijerph17186880

**Published:** 2020-09-21

**Authors:** Jami Eller, Jeffry A. Simpson

**Affiliations:** Department of Psychology, University of Minnesota, Minneapolis, MN 55455, USA; simps108@umn.edu

**Keywords:** attachment security, partner buffering, romantic relationships, context

## Abstract

Attachment insecurity is consequential for both personal and relationship wellbeing. Some research has documented that partner buffering can downregulate insecure individuals’ immediate feelings of distress, allowing them to feel more secure at least temporarily. The benefits of partner buffering, however, may be limited by several contextual factors. In this article, we identify boundary conditions that may curb or amplify the benefits of partner buffering for both targets (those who receive buffering) and agents (those who enact buffering). We suggest that motivation, ability, and timing may all affect partner buffering outcomes for targets and agents. If partner buffering is delivered in an adaptive way that does not reinforce the target’s insecure tendencies, it may help insecure targets learn that they can trust and depend on their partners (agents), which may facilitate greater security in targets. We recommend that future research consider these contextual factors and examine partner buffering as an inherently dyadic relationship process capable of enhancing attachment security.

## 1. Introduction

“For all of us, the person we love most in the world, the one who can send us soaring joyfully into space, is also the person who can send us crashing back to earth.” —Sue Johnson, Hold Me Tight: Your Guide to the Most Successful Approach to Building Loving Relationships, 2008 [1]

Romantic partners play a key role in influencing each other’s goals, emotions, and insecurities. One partner’s support and buffering (e.g., the tailored support and comfort they give to their distressed partners) can facilitate the other partner’s self-improvement [2,3], enhance relationship quality [4], and attenuate chronic attachment insecurities [5,6]. Along with work on couples receiving therapy [7,8,9], previous research with non-clinical couples indicates that better personal and relational outcomes can be achieved when partners buffer one another effectively [10].

However, just as partners can uplift and bolster each other, they can also induce unwarranted harm, even when they have good intentions. Thus, despite the benefits that partner buffering can offer, it may at times have unintended negative consequences for the target (e.g., the recipient of buffering) and/or the agent (e.g., the person doing the buffering). Indeed, recent research has documented that some positive behaviors, such as forgiveness, can lead to more negative intra- and inter-personal outcomes [11,12], whereas some negative behaviors, such as directly confronting problematic issues, can result in more positive outcomes [13]. These findings suggest that unique features of the individual and relationship may be just as important as the behaviors that targets and agents enact in determining their outcomes, both in the short-term and over time.

Limited theorizing and research have addressed the boundary conditions of partner buffering, including how such conditions may limit or increase its success. In this paper, we discuss the advantages and drawbacks of partner buffering along with the boundary conditions that may contribute to its effectiveness. We utilize partner buffering of attachment insecurity to demonstrate the buffering process, given the preponderance of partner buffering evidence in attachment research, but recognize that partner buffering is also relevant to other domains and constructs.

We begin by providing a brief overview of attachment theory, after which we review the existing literature on partner buffering. We then propose three general boundary conditions—motivation, ability, and time—that may determine the magnitude of benefits of partner buffering in many relationships. Notably, we focus on the consequences of buffering for the target (the person receiving the buffering) and agent (the person doing the buffering), the latter of whom has received much less consideration in previous research. We believe this dyadic perspective is imperative to understand this interdependent goal pursuit more fully [14].

## 2. Attachment Theory

Partner buffering has been most widely studied in relation to how individuals (agents) regulate the attachment-related needs of their partners (targets). Attachment theory addresses personality and social development in response to experiences with attachment figures across the lifespan [15,16,17,18]. Most relevant to partner buffering, attachment theory posits that individuals seek proximity to close others, desire a relational “safe haven” during stressful times, and use others as a secure base from which to pursue other important life goals and tasks [15,19,20]. In adulthood, romantic partners typically serve as the primary attachment figures who meet one’s attachment needs [21].

In addition to these normative functions of attachment, individuals develop internal working models of themselves and others based on prior experiences with their attachment figures, which guide their thoughts, feelings, and behaviors in attachment-relevant situations [16,22]. These models are continually shaped by experiences with attachment figures throughout life, and they change in response to significant events or relationship experiences [23,24]. 

Two attachment dimensions—anxiety and avoidance—underlie adult romantic relationships [25,26]. Avoidant attachment tends to result from insensitive or unavailable caregiving, which promotes self-protective deactivation strategies [20,27]. Individuals high in avoidance typically withdrawal from situations that demand intimacy or dependence and tend to mistrust close others [28,29,30]. Anxious attachment usually results from inconsistent, intrusive, or incompetent caregiving, which generates self-protective hyperactivation strategies [20,27]. Individuals high in anxiety tend to have a “negative filter” with regard to daily relational events, experience greater emotional volatility, and have chronic concerns about the availability of their attachment figures [15,31,32]. Anxiety, as we discuss it throughout this paper, refers specifically to attachment-related anxiety and not to clinical anxiety. Individuals who score low in avoidance and anxiety tend to be secure. Secure attachment results from receiving consistent and appropriately sensitive caregiving, which allows individuals to develop more constructive, problem-focused strategies [20]. Secure individuals typically have the most positive relational and personal outcomes [33]. Despite this, all individuals experience moments of insecurity that compel them to seek proximity to, and reestablish a safe haven with, their attachment figures [15,19]. Effective partner buffering serves these first two functions—proximity seeking and safe haven—in individuals involved in adult romantic relationships.

## 3. The Current State of Partner Buffering Research

Romantic relationships often act as a medium through which individuals can be comforted when they feel threatened or upset (i.e., seeking proximity and safe haven) and can grow once threats have abated (i.e., secure base) [34]. Although relationship researchers have examined important ways in which individuals grow and change in relationships [35,36,37], partners must first feel safe and secure before they can grow [16,18]. Cast another way, momentary feelings of insecurity need to be mitigated before growth and possible long-term changes in attachment orientations can occur [38]. Partner buffering can mitigate these momentary afflictions. 

Partner buffering is a specialized form of caregiving most often employed by a romantic partner. It has been defined as supportive behaviors or actions that one partner (the agent) engages in to downregulate the emotional distress in their partner (the target) [10,39]. Partner buffering includes various types of support behaviors (e.g., emotional support, problem-solving advice, invisible support), so it is not limited to one or even a few types of behavior intended to downregulate targets. Partners, therefore, can serve as external emotion regulators. Just as self-regulation involves a wide variety of strategies [40], so too does partner buffering [10,38,39]. Partner buffering also encompasses a wide array of behaviors that support and downregulate partners in personalized, tailored ways. For example, highly anxious individuals tend to be buffered more effectively by emphasizing their relational values and strengths, whereas highly avoidant individuals are best buffered more instrumentally by focusing on their independence and autonomy [38,41]. At present, there is no evidence to suggest that partner buffering is more effective at low levels of target attachment insecurity than at high levels of target insecurity, which suggests how effective buffering can be if employed correctly. 

Partner buffering behaviors, however, must be tailored to certain features of partners, such as the target’s attachment orientation, as well as features of their specific relationship, such as its norms and overall quality. For example, even though emphasizing relational strengths may benefit anxiously attached individuals in general, such efforts may be ineffective in relationships characterized by low commitment [42,43,44,45]. Furthermore, partner buffering that is perceived by targets as inauthentic or counter-normative within the relationship often leads to amplification, rather than downregulation, of attachment insecurities [46,47,48]. Accordingly, partner buffering is an *inherently dyadic, idiosyncratic relationship process* in which behaviors intended to downregulate the target must be chosen judiciously and executed well to match the target’s and the relationship’s unique characteristics. 

Despite these idiosyncrasies, partner buffering has a fairly substantial literature revealing its effectiveness at improving both personal and relationship wellbeing, especially in the short-term. Perceiving that a partner is available during a stressful event, in and of itself, can lessen emotional burdens and feelings of insecurity [49]. Indeed, merely priming mental representations of a partner can temporarily reduce negative affect and feelings of insecurity [50]. In other words, simply being reminded of one’s partner can provide comfort and be beneficial, even when partners are not physically present during a stressful time. When partners are physically available, perceiving them as responsive and sensitive to one’s needs attenuates negative emotional escalation and facilitates personal coping [19,51,52]. Although positive representations and responsive behaviors are broadly beneficial, certain critical characteristics of targets—especially their form of attachment insecurity—is likely to determine how effective different types of partner buffering will be. 

Anxious individuals, for example, are buffered more effectively when their partners meet their needs for greater closeness, interdependence, and felt security [38]. Tran and Simpson [45], for example, found that anxious targets behave in more constructive and deescalated ways with their romantic partners when anxious targets have partners who report higher relationship commitment. Tangible signals of partner affection also tend to be effective for anxious targets. An affectionate touch from a partner and their sexual satisfaction, for instance, are beneficial to anxiously attached individuals’ emotional and relational wellbeing [53,54]. With anxious targets, however, these behaviors must offer reassurance and validation while not exacerbating anxious targets’ escalated emotions [38]. This is a fine line to walk, particularly in stress-inducing situations.

Avoidant individuals are buffered more effectively when partners meet their needs for independence, autonomy, and personal control, while still conveying their availability [38]. Practical/instrumental support from partners tends to be more beneficial to avoidantly attached individuals’ personal and relational wellbeing [41,55,56]. In general, avoidant targets’ sense of autonomy/control is less threatened when they receive practical or instrumental versus emotional support. Avoidant targets also tend to be buffered more effectively during conversations when partners do not make additional requests or impose burdens, but are instead are carefree. Positive and light-hearted exchanges that involve play or gratitude, for example, are particularly beneficial for the personal and relational wellbeing of such individuals [57,58]. Nonverbal affection that does not involve physical touch, but still conveys availability, is also associated with more positive affect and behavioral receptiveness for highly avoidant individuals [59]. With avoidant targets, therefore, it is important to provide space and autonomy while also providing consistent signs of availability [38], which also is a fine line to walk.

In most previous research, the documented effects of partner buffering have been short-term, limited by cross-sectional designs. In examining the effects of partner buffering over time, however, Arriaga and colleagues [5] found that the effects of partner buffering vary depending on the timing of the outcome assessed. Specifically, individuals who perceived more comfort from their spouse during a stressful life event (e.g., the transition to parenthood) reported lower attachment anxiety at the same assessment, but comfort did not predict lower attachment anxiety at the following assessment six months later. Similarly, in a study of couples attempting to induce a change in their partner, Overall and colleagues [13] found that when individuals engaged in more indirect behaviors (e.g., covert, passive influence strategies), they believed the interaction was more successful in the short-term. Three months later, however, these indirect behaviors did not predict positive movement toward the goals they had discussed with their partner three months earlier. Collectively, these findings suggest that partner buffering most likely serves an immediate function—to attenuate the target’s current emotional upheaval.

Although this initial research is important, no partner buffering research to our knowledge has addressed the conditions underlying the success or failure of immediate partner buffering attempts. The current research simply suggests that when partners engage in certain types of behavior, many targets tend to benefit. However, we do not know why buffering does not always occur when targets are distressed, when it is less effective when enacted, and what the residual side effects of buffering are for both targets and agents. We need to identify what these boundary conditions are to achieve a better, more nuanced understanding of partner buffering and eventual security enhancement. We propose that the boundaries of partner buffering can be conceptualized most clearly by considering the target’s, as well as the agent’s, motivation, ability, and timing.

## 4. Boundary Conditions of Partner Buffering for Targets

Targets of partner buffering have been widely investigated, and they typically benefit from buffering. This conclusion is reasonable, given that targets should inherently gain when they receive good, well-tailored support. Targets of buffering frequently experience increases in self-esteem [60,61], improved relationship quality [54,62], and elevated attachment security [5,6,63]. However, the effects of buffering on targets may not always be positive or clear-cut. Much of the previous buffering research has assumed that targets are motivated to change or improve and that they have communicated the need for buffering (e.g., they have conveyed visible signs of distress or have made verbal requests for support). With a few notable exceptions [5], prior research has not examined the sensitivity and contextual nature of partner buffering processes over time. We propose that three conditions—motivation, ability, and time—might largely determine the success or failure of partner buffering and could partially explain why most partner buffering effect sizes tend to be small-to-moderate in magnitude.

### 4.1. Motivation

An unstated premise in many partner buffering models is that targets are, at some level, motivated to be downregulated and to reduce their current level of distress [10,38]. Although this is likely to be true of many targets, some targets may not want their partners to act as external emotion regulators or facilitators of change. Conversely, other targets may want their partners to buffer them continually, remaining unmotivated to change or develop their own regulation skills [64,65]. The motivation to want, to resist, or to be overly reliant on partner buffering should affect the effectiveness of an agent’s buffering attempts.

In some instances, targets may view attempts by their partner to buffer their emotions as infringing on their sense of autonomy or personal competence [66]. Although having a partner who supports one’s autonomy needs tends to be beneficial [67], some targets—particularly those high in avoidance—may be motivated by very high and perhaps relationship damaging desires for autonomy. Avoidant targets’ prior attachment experiences may have instilled a strong self-protective motivation to maintain autonomy and avoid rejection from unavailable or reluctant caregivers [16,68]. Within their romantic relationships, avoidant targets may continue to act on these motivations by responding negatively to their partner’s attempts to provide direct and especially emotional forms of support [41,69]. Indeed, when their partners try to establish greater closeness when avoidant targets are distressed, avoidant targets typically withdraw [70]. This pattern of behavior by avoidant targets inhibits partners from being able to employ buffering behaviors. For avoidant targets, therefore, finding the right balance of autonomy with small introductions of dependence is necessary in order for buffering to be successful [38]. If agents employ effective “soft” buffering techniques, avoidant targets should not recognize that they are being buffered. Although this may provide temporary relief, the motivation of the target to seek autonomy in the future is not likely to change despite the agent’s attempts to employ effective buffering, which should sustain avoidance. As a result, avoidant targets should not experience long-term changes in avoidance unless they realize that greater interdependence is safe and positive, and they are motivated to achieve it.

Targets on the other end of the spectrum may be overly reliant on their partners and lack the motivation to regulate themselves when they are distressed. Although most people resist being over-benefitted in relationships [71,72], some develop intricate overdependence on their partners and relationships [64,65,73]. Highly anxious targets, for example, can become over-dependent on their caregivers [16,74,75]. In addition, they are sensitive to changes in the perceived availability and regard of their partners [61] and engage in behaviors to elicit more closeness with them, even when their own personal needs and motives should take precedence [75]. This pattern of dependence motivation and prioritizing relationship goals above personal needs may lead anxious targets to rely heavily on their partners to buffer their negative emotions instead of developing an autonomous set of emotion regulation abilities. This pattern, in turn, should be detrimental to their personal development and growth, which might maintain their high level of anxiety.

A target’s motivation for receiving partner buffering might also affect the effectiveness of partner buffering, as well as the target’s future personal growth. Avoidant targets motivated by autonomy and control may resist most buffering attempts or stop partners from engaging in buffering behaviors once they begin. Anxious targets motivated by interdependence may become overly dependent on partner buffering and never develop their own emotion regulation skills. Even though these motivations are well-studied and understood, more attention needs to be dedicated to examining precisely how agents tailor their buffering to the unique attachment needs and motives of their target partners. Moreover, future research should attempt to manipulate the motivations of insecure agents before they engage in a support interaction with a target to determine whether alterations in an agent’s motivation result in improved outcomes for the target.

### 4.2. Ability

A target’s downregulation of emotions by their partner (the agent) may also depend on the target’s ability to signal that they need buffering. Early in life, children learn to signal or indicate when they need a caregiver’s attention [27,76]. In infancy, this involves crying, screaming, cooing, or approaching a caregiver to achieve proximity and safe haven functions [14]. Signaling distress in adulthood, however, is often more complex. Effective signaling in adulthood requires targets to recognize their distress and then calibrate how to express their need for buffering to their partner in a clear, socially appropriate manner.

Some targets may struggle to recognize their level of distress or its true source. Insecure targets, for example, may lack emotional awareness or clarity [77,78], which might inhibit them from signaling their actual needs to their partner in a clear, effective manner. Additionally, some targets may not begin signaling their needs until their distress and negative emotions are high. At this point, it may be difficult for targets to communicate effectively and for their partners to buffer them well.

Support-seeking behaviors provide some insights into how distress is communicated to partners (agents). When targets seek support, they may do so through explicit requests for specific types of support [79,80] or through subdued or subconscious solicitation of support [81]. In addition, the effectiveness of explicit requests for support varies depending on the content of the request. More effective requests for support from targets involve constructive conversations that emphasize problem-solving rather than burdensome criticism or complaints [3,69]. Support-seeking that is clear and positive typically elicits more positive support from partners, which improves the likelihood of resolving problematic issues. When anxious and avoidant targets are most in need, they often do seek support [6,24,69,70], but less effectively than their secure counterparts.

However, when targets are distressed and need buffering, signaling their need for support in constructive ways may be difficult or even paradoxical. Distressed targets require buffering to downregulate their emotions. Before they can be downregulated, however, anxious targets often experience heightened negative emotions, whereas avoidant targets frequently withdraw or disregard the negative emotions they are experiencing [20]. Neither escalation nor withdraw/denial is a positive approach for requesting support. The paradox is that needing to be downregulated in order to signal the need for partner buffering is likely to inhibit sufficient signaling behavior in insecurely attached individuals.

A target’s ability to recognize and then signal their distress may also significantly influence whether and how partner buffering processes unfold. For example, targets who are unable to recognize their distress soon enough may become too emotionally escalated for effective downregulation by the time their partners start buffering. However, even among targets who do recognize their distress, adequately signaling that they need to be buffered presents another paradox since support-seeking tends to be most successful when targets are calm and require less buffering. In sum, the ability of targets to recognize their distress and signal their need for buffering may largely determine both the receipt and effectiveness of partner buffering from agents. Future research should emphasize coding signaling behaviors during buffering interactions to examine (a) which, if any, signaling behaviors are most commonly displayed by insecure targets, and (b) which signaling behaviors are most often met with appropriately tailored support from agents.

### 4.3. Time

Targets are not stagnant across time, nor is the partner buffering they receive. Growing evidence suggests that major life and relationship events can shift attachment orientations over time if key attachment needs are met [4,24,82]. When these changes occur, partner buffering should be most impactful in the immediate moment, whereas other features of personal growth associated with secure base behaviors should be operative across longer periods [5]. Some degree of partner buffering, however, is still required across time, but it must be expressed in tailored, adaptive ways.

In order to be effective across time, adapting partner buffering is likely to be necessary for targets to experience greater attachment security while remaining downregulated [38]. To accomplish this, agents may need to provide partner buffering in a scaffolded manner over time. Specifically, when felt insecurity is high in targets, greater buffering may need to be enacted by agents, but as the target’s general level of attachment security increases, they should require less partner buffering in most distressing situations. With such scaffolding, targets may still experience setbacks that require temporary upticks in buffering, necessitating that agents remain adaptive and alert to the target’s shifting signals and needs. At present, no established guidelines exist regarding how to best scaffold romantic partners across time as their level of attachment security increases or decreases.

One technique relevant to the long-term adaptability of a target’s needs for partner buffering is just-in-time interventions. Health psychologists have developed these interventions, which provide real-time feedback and scaffolding in support as a partner’s health conditions progress and change [83]. For example, one just-in-time intervention tracks computer activity. It prompts users to stand up and walk, but only when there has been consistent computer activity for more than 30 minutes. These tailored interventions work by identifying the optimal time during which targets are most in need of support and may be most receptive to it [84]. Although such health interventions rely on technology as the intervening technique, the application to time may prove useful in promoting better partner buffering effects for targets.

Optimal times for targets to receive partner buffering as they change in attachment security could be identified by considering a target’s specific support needs along with their receptivity in much the same way as just-in-time interventions do. For example, early in the process when a target’s insecurity is still high, there should be more times when partner buffering will be helpful. But as the target’s security increases, the number of optimal times should decrease as more of the target’s needs are being met due in part to their more secure working models. Across time, this scaffolding approach, which is based on both the needs and receptivity of targets, should sustain or improve the personal and relational wellbeing of most targets. It should do so by allowing targets to continue realizing that their partners continue to be available safe havens, while also allowing for adaptive, attachment-relevant tailoring that promotes effective partner buffering. Future research should continue to employ diverse longitudinal methods—such as momentary ecological assessments, signal-response methods, and daily diaries—to capture the optimal timing of partner buffering.

## 5. Boundary Conditions of Partner Buffering for Agents

Partners (agents) also play an integral role in the partner buffering process, but they have received much less attention, both empirically and theoretically. The image of an agent who is able and willing to engage in adaptive, well-tailored partner buffering both consistently and over vast stretches of time is alluring. To the extent that agents can perform this optimal and effective form of partner buffering, some have suggested that partner buffering “…ultimately yield[s] benefits for both partners as well as their relationship” [85] (p. 7). Indeed, there are numerous benefits of giving support, affection, and gifts to partners, such as feeling more positive emotions [86,87], reporting less stress [88,89], having increased self-worth [90,91], and experiencing improved relationship quality and closeness with the support recipient [92]. Recognizing that providing affection and support can have many beneficial outcomes, these effects should also be bounded by contextual factors surrounding such behaviors [93]. Similar to investigations involving targets, most prior research has overlooked contextual factors and assumed that agents are typically motivated, able, and willing to engage in fine-tuned partner buffering, neglecting the integral role of time in these complex processes.

### 5.1. Motivation

An agent’s general and specific motivation to enact partner buffering is likely to affect in part how effective their buffering will be. According to interdependence theory [94,95], as partners develop their relationship, many experience a transformation of motivation in which their motives shift from being self-oriented to being more partner- and relationship-oriented. This increased communal motivation tends to improve both personal and relational wellbeing [96,97]. However, agents may differ in how motivated they are to foster interdependence and aid targets in pursuit of security, which could affect their engagement in buffering behaviors [14].

Not all relationships, however, are communal relationships, and some partners do not possess communal motivations. Some people develop and maintain relationships with partners that are exchange-oriented, defined by a transactional account of receiving and giving benefits between partners [98,99]. In exchange-oriented relationships, partners feel indebted to one another after receiving a benefit. If, for example, an agent buffers a target (their partner), the target is likely to feel indebted for the successful downregulation until the target can return some benefit to the agent. In this case, the agent’s initial motivation for providing care is not necessarily out of selfless concern for the target, but rather due primarily to a desire to maintain an equitable, balanced relationship. This may result in less effective partner buffering, which might be perceived by targets as inauthentic.

Moreover, not all people harbor positive, other-oriented motivations in all situations or relationships [100,101]. An agent who lacks other-oriented motivation, for instance, may be unwilling to enact any buffering behaviors whatsoever. On the other hand, if more self-oriented agents engage in partner buffering, their actions may lack the authenticity and fine-tuned tailoring that is likely to be enacted by other-motivated agents.

Even within strong communal relationships, of course, some amount of reciprocity across time is still necessary. Lack of reciprocity in communal motivation, along with minimal caregiving and buffering, does not allow partners to take full advantage of relationship benefits, which are essential for remaining in satisfied, committed relationships over time [94,95]. In a large meta-analysis, Le and colleagues [97] found that relationship partners’ partner-specific communal motivations tend to be moderately correlated (*r* = 0.26), but it explains a rather small percentage of the variance in partners’ shared communal motivation. This leaves substantial room for partnerships in which one partner (e.g., the agent) might be more or less communally motivated than the other (e.g., the target). Accordingly, agents who are consistently buffering, but not receiving similar care in return, may decrease in their communal motivation or experience caregiver burnout over time [97,102,103].

Finally, the degree to which agents’ motivation to engage in partner buffering is intrinsic or extrinsic might also affect buffering outcomes. Partners (agents) who give intrinsically motivated support more frequently also tend to render effective support and benefit more from it [93,104]. However, when agents are less intrinsically motivated or feel obligated to give support, it is less effective and more costly personally. If, for example, an agent chooses to buffer because they do not want the target to be upset or feel distressed, they may be more likely to display adaptive, authentic behaviors when buffering. However, if an agent feels obligated to buffer because the target explicitly requests it or someone else (e.g., a therapist) insists on using specific communication skills, they might be less engaged, display more limited buffering behaviors, or appear less authentic when enacting them.

In sum, the motivations underlying partner buffering ought to influence whether buffering occurs and, if so, how effective it is. Partners (agents) who are communally motivated, show reciprocation in communal motivation over time, and are intrinsically motivated should be more effective at buffering the target’s emotions and ought to experience the personal benefits associated with giving to others. Not all partners, however, are likely to meet these criteria. When they do not, the effectiveness and influence of partner buffering should be attenuated, and these agents are likely to experience more negative consequences when attempting to buffer targets. Future research should manipulate agents’ communal motivation and examine how such manipulations alter the displays and effectiveness of their buffering behaviors.

### 5.2. Ability

Partner buffering may also be limited by an agent’s ability to buffer. Most current models of partner buffering assume that the majority of agents will typically attempt to engage in buffering behaviors when targets are distressed [10]. Indeed, Lemay and Dudley [105] found that partners often adapt their own emotions and displays of affection when they have an insecure partner. This may represent an adaptation of sorts while enacting partner buffering behaviors. Yet, despite this immediate benefit to the target, Lemay and Dudley [105] also found that when agents adapted their emotions and behaviors to compensate for the target’s insecurity, agents reported declines in relationship wellbeing. That is, even though agents can adapt to meet the target’s needs, these adaptations may be costly for the agent.

Even when agents are willing and appropriately motivated to enact buffering, their attempts might still fail if the target’s emotional upheaval is too severe. As discussed earlier, effective partner buffering requires agents to recognize a target’s signals of distress, skillfully determine which buffering behaviors to use to soothe the target in the specific situation, and then adapt and alter these behaviors across time. This is a tall order even for the most proficient and secure partners in stable, committed relationships. Additional barriers to this undertaking, such as partners’ incompatible attachment orientations, needs, or motives, may make engaging in effective partner buffering even more challenging.

In general, agents who are insecurely attached may be less able and/or willing to engage in effective partner buffering. Individuals high in attachment anxiety, for example, experience greater emotional and physiological stress when faced with potential conflicts with their partners [19,31,106]. They also tend to lack self-regulation and emotion regulation abilities to downregulate their negative emotions when they are upset [68]. Such emotional upheaval may inhibit anxious agents from recognizing the distress that their partners (targets) are experiencing. If, however, anxious agents can effectively manage their negative emotions during stressful situations and recognize the target’s distress, they still face the challenge of responding to the target in sensitive, tailored ways. Prior research has revealed that anxious individuals are more likely to interact with their partners in ways that are damaging, domineering, and hostile [71,107,108]. Predictably, anxious agents also struggle to provide effective support to targets, even when they are able to keep relationship destructive behaviors at bay [69]. This combination of destructive behaviors and reduced ability to enact appropriate support runs counter to the basic requirements of effective partner buffering.

Highly avoidant individuals, on the other hand, are inclined to withdraw and disengage during stressful or personally threatening situations [68,108,109]. Avoidant individuals who are disengaged tend to pay less attention to their partners and, therefore, may fail to recognize their partner’s distress signals. Yet even if avoidant agents can engage and recognize a target’s distress, they still must respond in sensitive, tailored ways. Similar to anxious agents, avoidant agents also struggle with providing adequate support to their partners (targets) [70,108,109], and they are more likely to respond aggressively when they feel stressed [109,110,111]. Thus, much like anxious individuals, the combination of poor support-giving and the inclination toward destructive behavior should typically undermine effective partner buffering by avoidant agents.

In sum, an agent’s ability to recognize a target’s distress and then engage in appropriate buffering behaviors should affect whether buffering occurs and how effective it ultimately is. Providing effective partner buffering is a complex and difficult process, even without factoring in the unique features of the two partners and their relationship. For insecurely attached agents, however, recognizing a target’s distress and engaging in effective buffering should be especially challenging. Future research should examine how an agent’s extra-dyadic resources—such as their social networks of friends or family members—may assist them in being able to identify the target’s distress and provide effective buffering in response to it.

### 5.3. Time

Finally, effective partner buffering by agents may be influenced by the stage of development of a close relationship, by events that continually reoccur over time, or by important interactions that partners have. Given that it inherently is a dyadic, idiosyncratic process, effective partner buffering should take time to develop, it must be tailored to the needs, desires, and expectations of the target and/or relationship, and it must remain adaptive as events change following significant interactions.

Beginning a relationship with a partner involves an initial period of getting to know one another and establishing norms and expectations within the relationship [112]. During this initial period, partners become more familiar with each other. Although some partner buffering is likely to take place early on, it may be less frequent or less effective than it will be later on because, across time, relationship development generates greater interdependence, shared goal pursuit, and the eventual emergence of effective buffering [14]. The rate of partner knowledge acquisition and familiarity is also likely to vary across couples, revealing the unique trajectory that partner buffering follows in a given relationship [113,114]. Over time, however, most partners should gain enough knowledge about one another to develop tailored buffering strategies consistent with relationship norms and expectations, depending on the agent’s and the target’s specific motivations, needs, and abilities.

As the relationship develops, partners may also differ in how frequently they enact buffering behaviors relative to each other. If this happens, partners may become aware of this inequity in their relationship, particularly if it occurs over long periods of time in which one partner is consistently over-benefitted or under-benefitted [72]. Partners who are consistently under-benefitted (e.g., an agent who always buffers a target without reciprocity) may begin to feel underappreciated [71] or burnout [97,103]. Thus, one cannot assume that agents can (or should) be willing to buffer targets repeatedly across time without having their own needs being met as well. Such inequality should also not benefit targets over time if their partners (agents) lose the motivation or willingness to buffer and the relationship unravels. Future research should continue to investigate partner buffering longitudinally to document how buffering develops and is reciprocated over time and how variations in buffering across time affect specific outcomes for both agents and targets.

The long-term effects of partner buffering on agents aside, interactions in which buffering occurs are likely to be dynamic. Consider a target who is distressed and signals their need for comfort and support. Imagine first an interaction in which an agent is consistently supportive of the entire conversation, regardless of what the target says, feels, or does during the interaction. On the surface, this might appear to be good partner buffering, given that the goal is to downregulate the target’s negative thoughts and feelings. Now imagine the same interaction in which the agent is consistently supportive, but the agent also adapts (alters) their behavior in response to what the target says, feels, or does in an attempt to convey support. Although the first scenario entails supportive actions, the agent’s behavior may be insensitive to the changing needs or expectations of the target as the conversation ebbs and flows. The second scenario, on the other hand, may reflect the agent’s responsiveness to the changing needs of the target as well as the changing flow and pattern of the conversation. The agent in the second example, in other words, may be enacting a constellation of more adaptive partner buffering behaviors rather than a single, less responsive, unwavering display of support. It is this adaptability to the target and the immediate moment that may characterize more effective forms of partner buffering. Future research should utilize interval coding in order to assess the nuanced exchange and adaptability of partner buffering behaviors in diagnostic relationship interactions.

## 6. Conclusions

In conclusion, partner buffering can improve personal and relational wellbeing and should play a role in enhancing attachment security over time. However, one must weigh not only the potential benefits of partner buffering, which have been widely investigated, but also its potential costs to the target and the agent. Partner buffering that is well-tailored to the characteristics and needs of the target and the relationship can, and frequently does, downregulate targets effectively when they are distressed. However, the benefits for targets and agents are likely to be constrained by several important contextual factors. We suggest that motivation, ability, and time may act as boundary conditions that either decrease or increase the effectiveness, as well as the benefits, of partner buffering.

Future research should examine the target’s motivation to be buffered along with their skill at signaling their need for buffering. Research should also address the agent’s motivation to buffer in conjunction with their ability, even if they are intrinsically motivated to buffer targets well. Finally, research should explore how partner buffering develops and unfolds across time in different types of relationships and should examine additional outcomes. Answers to these issues will advance our understanding of not only how relationships are maintained, but how attachment security might be enhanced in part through partner buffering processes.
